# The P Balasubramaniam Award-2024 Singapore Orthopaedic Association Annual Scientific Meeting Award: Novel artificial intelligence algorithm for soft tissue balancing and bone cuts in robotic total knee arthroplasty improves accuracy and surgical duration

**DOI:** 10.1186/s42836-025-00322-1

**Published:** 2025-08-04

**Authors:** Matthew Song Peng Ng, Ryan Wai Keong Loke, Melvin Kian Loong Tan, Yau Hong Ng, Zi Qiang Glen Liau

**Affiliations:** 1 Department of Orthopaedics, National University Hospital, National University Health System, Singapore, Singapore; 2 Department of Orthopaedics, Alexandra Hospital, National University Health System, Singapore, Singapore; 3 Yong Loo Lin School of Medicine, National University of Singapore, National University Health System, Singapore, Singapore

**Keywords:** Robotic, Total knee arthroplasty, Arthroplasty, Joint replacement, Orthopaedic surgery

## Abstract

**Background:**

Robotic Total Knee Arthroplasty (rTKA) has become increasingly popular. Intraoperative manual planning of femur and tibia implant positions in all degrees of freedom to achieve surgeon-defined targets and limits of bone cuts, gaps, and alignment is challenging. The final manually defined solution may not be optimal, and surgical duration increases significantly. We aim to demonstrate the effectiveness of our novel algorithm in terms of accuracy and surgical duration.

**Methods:**

We developed a novel AI computational algorithm to optimize rTKA implant positioning in three-dimensional space. The initial parameters of 3D implant positioning and surgeon-defined target gaps and bone cuts are set. The algorithm determines permutations achieving ideal 3D implant positioning with ± 0.5 mm accuracy, ranking them by surgeon preference and evidence-based criteria. We compared accuracy in achieving surgeon-defined target gaps, intraoperative soft tissue balancing duration, and total surgical time.

**Results:**

A prospective study of 67 consecutive rTKA patients at a tertiary institution (Nov 2021–Dec 2023) was conducted. 25 patients (mean age 70.4 ± 7.34 years) had our algorithm used intraoperatively, while 42 (mean age 70.5 ± 6.90 years) did not. 92% of rTKAs using our algorithm achieved target gaps ± 1.5 mm, vs. 52% of non-algorithm rTKAs (*P* = 0.003). The average difference between surgeon-defined target gaps and final achieved gaps was 1.1 ± 0.5 mm in the algorithm group vs. 1.8 ± 1.0 mm in the non-algorithm group (*P* = 0.003). Soft tissue balancing duration was significantly shorter: 1.16 min ± 0.11 with algorithm use vs. 14.5 min ± 8.3 (*P* < 0.0001). Total surgical duration was also significantly lower: 38.4 min ± 14.9 vs. 73.7 min ± 19.6 (*P* = 0.0002).

**Conclusion:**

Our novel AI algorithm significantly improves accuracy in achieving surgeon-defined target extension and flexion gaps while reducing soft tissue balancing and total surgical duration. This is highly promising for achieving both reproducibility and efficiency in rTKAs.

Video Abstract

**Supplementary Information:**

The online version contains supplementary material available at 10.1186/s42836-025-00322-1.

## Introduction

The total knee arthroplasty (TKA) surgery is one of the most commonly performed joint surgeries in the world, with recent data showing 82% of TKAs survive till 25 years [1]. Unfortunately, TKAs fail, apart from acutely due to infection, in the long run due to malalignment, loosening, and instability [2]. Furthermore, up to 20% of patients are dissatisfied post-TKA [3]. Poor patient-related outcomes have been closely associated with implant malalignment [4]. Hence, robotic total knee arthroplasties (rTKA) have been gaining in popularity over the past few years, with numerous studies claiming better implant positioning and superior mechanical and anatomical alignment while having similar clinical and functional outcomes [5, 6]. This has led to the rapid development of many robotic systems for rTKAs, including the ROSA Knee system by Zimmer Biomet [7], the CORI system by Smith and Nephew [8] and the MAKO Total Knee system by Stryker [9].

**Fig. 1:**
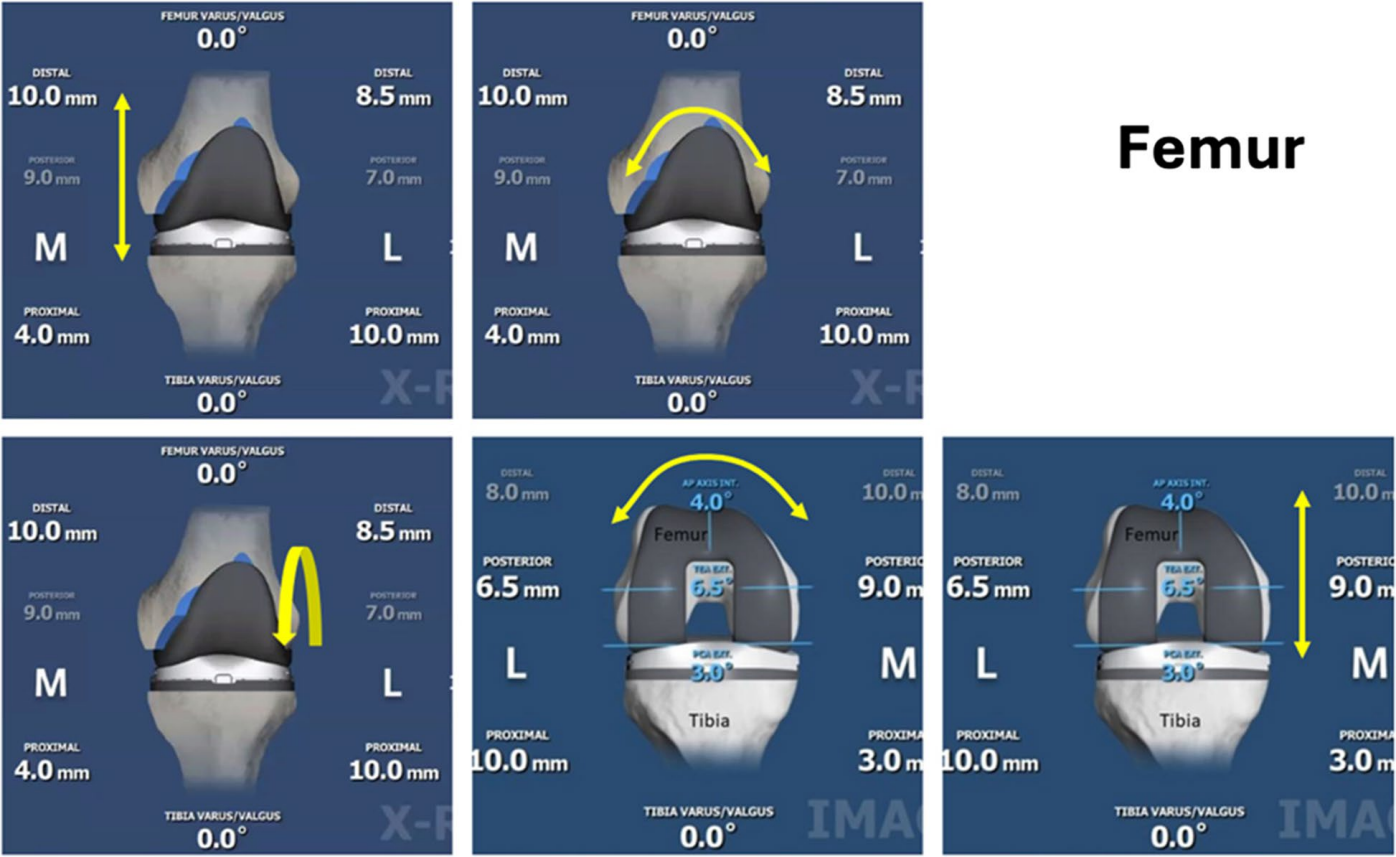
Femoral implant degrees of freedom. (**A**–**B**, top row: left to right; **C**–**E**, bottom row: left to right) (**A**) Proximalization/Distalization; (**B**) Varus/Valgus Adjustment; (**C**) Flexion/Extension; (**D**) Internal/External Rotation; (**E**) Anteriorization/Posteriorization

**Fig. 2:**
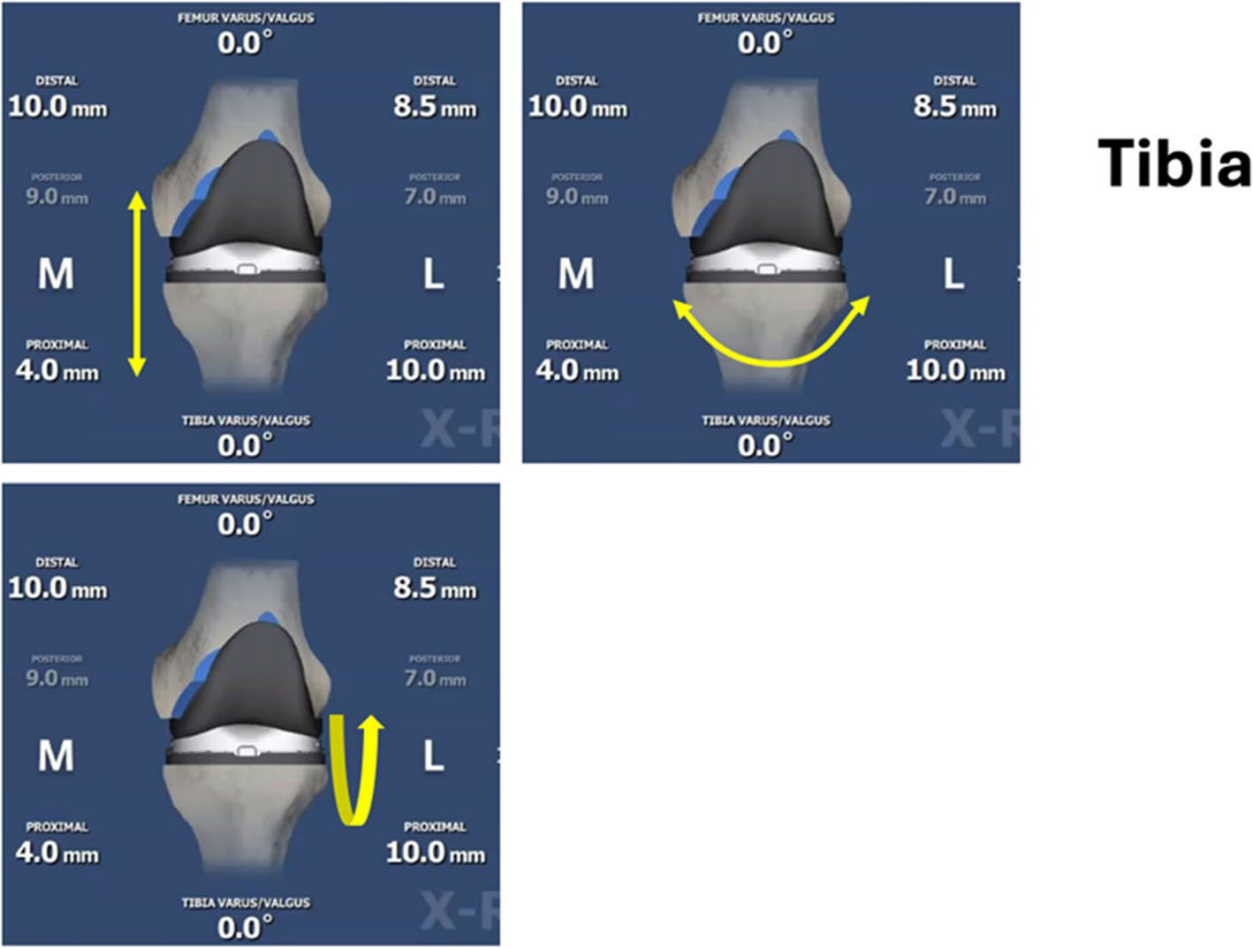
Tibial implant degrees of freedom. (**A**–**B**, top row: left to right; C, bottom row: left). (**A**) Proximalization/Distalization; (**B**) Varus/Valgus Adjustment; (**C**) Flexion/Extension;

Regardless of which robotic system is used, there will always be a planning step during the rTKA process before actual bone cuts are made, where the surgeon needs to manually input a set of parameters to position the femoral and tibial implants with the knee in 0 degrees and 90 degrees [10]. For instance, the ROSA Knee system allows the surgeon to manually translate and rotate both the femoral and tibial implants separately in the coronal plane when the knee is extended, translate the femoral implant in the sagittal plane when the knee is flexed, and rotate the femoral implant along the posterior condylar axis when the knee is flexed. The ROSA Knee system then displays real-time, the resulting bone cuts, bone gaps, and soft tissue gaps in both flexion and extension views (Figs. 1 and 2).

This step aims to achieve a final result that balances the knee and aligns as closely as possible to the surgeon’s alignment philosophy. Currently, there exist numerous alignment philosophies [11]. Traditionally, the femoral and tibial components are aligned perpendicular to the mechanical axis of the lower limb [12], keeping the hip-knee-ankle (HKA) angle and the joint line at neutral. However, this disregards the fact that among individuals, there are significant constitutional differences in the HKA and the joint line obliquity [13], which resulted in the creation of kinematic alignment that aims to achieve the native alignment of the pre-arthritic knee [14]. Lately, more philosophies have been published in literature, including inverse kinematic alignment [15], functional alignment [16], restricted kinematic and restricted inverse kinematic alignment. While these philosophies are still being extensively debated, there is no clear consensus on which is the superior philosophy to use [17], and it is completely up to the surgeon’s preference. To add to the assortment of differing philosophies, there exists considerable intra-observer surgeon variability in employing the steps to achieve a range of targets that he has in mind.

However, since implants can be individually translated and rotated in multiple planes and different axes, this creates a unique problem. Since the implants can be adjusted in so many degrees of freedom, thousands of unique alignment solutions exist for each rTKA case, and it is nearly impossible for the surgeon to manually run through all possible solutions systematically to consider which solution best suits their alignment philosophy and personal tolerances. Frequently, the surgeon ends up taking an extended period of time settling for a solution that may not always be the most optimal, or fulfill the alignment philosophy they are trying to achieve.

Hence, our team has created a novel artificial intelligence (AI) computational algorithm that can quickly compute the set of parameters that the surgeon can input to achieve the optimal positioning of the implants in three-dimensional space, based on the surgeon’s preferred philosophy and constraints. Currently, no existing algorithm systematically assists in exploring and optimizing all possible implant positioning solutions, making our proposed approach potentially valuable in addressing this gap. Our study aims to demonstrate the effectiveness of utilising this novel algorithm clinically in terms of soft tissue balancing accuracy and surgical duration. We hypothesise that our novel AI algorithm achieves higher soft tissue balancing accuracy and requires shorter surgical and planning duration in ROSA rTKAs.

## Methods

### Patient selection

This is a prospective, consecutive series that included two distinct groups of patients undergoing rTKA with the imageless ROSA System by Zimmer Biomet, between November 2021 and December 2023. The first group comprised patients whose procedures utilized a novel AI algorithm designed to optimize implant positioning. All surgeries in this group were performed exclusively by a single surgeon. The second group consisted of patients who underwent rTKA without the use of the novel algorithm. These surgeries were performed by two other surgeons at our institution.

In each group, patients were selected consecutively based on their presentation to the respective surgeons during the study period, provided they met the inclusion and exclusion criteria defined for this study. Inclusion criteria included a) patients undergoing rTKA for Primary Knee Arthritis; b) Male and Female Non-Pregnant patients aged 21 and above; c) willing to undergo rTKA with ROSA Knee System by Zimmer Biomet. Patients were excluded if they involved a) complex TKA cases such as Revision TKA/UKA or Conversion TKA from Prior Tibial/Femoral Osteotomy; b) Arthrodesis of Affected Joint; c) Active or suspected infection in or about the knee joint.

### Novel algorithm

The algorithm was conceptualised and developed entirely in-house, independent of ROSA or any robotic TKA companies, before being packaged into a standalone application with a Graphical User Interface (GUI) that can run on any computer without an internet connection.

**Fig. 3:**
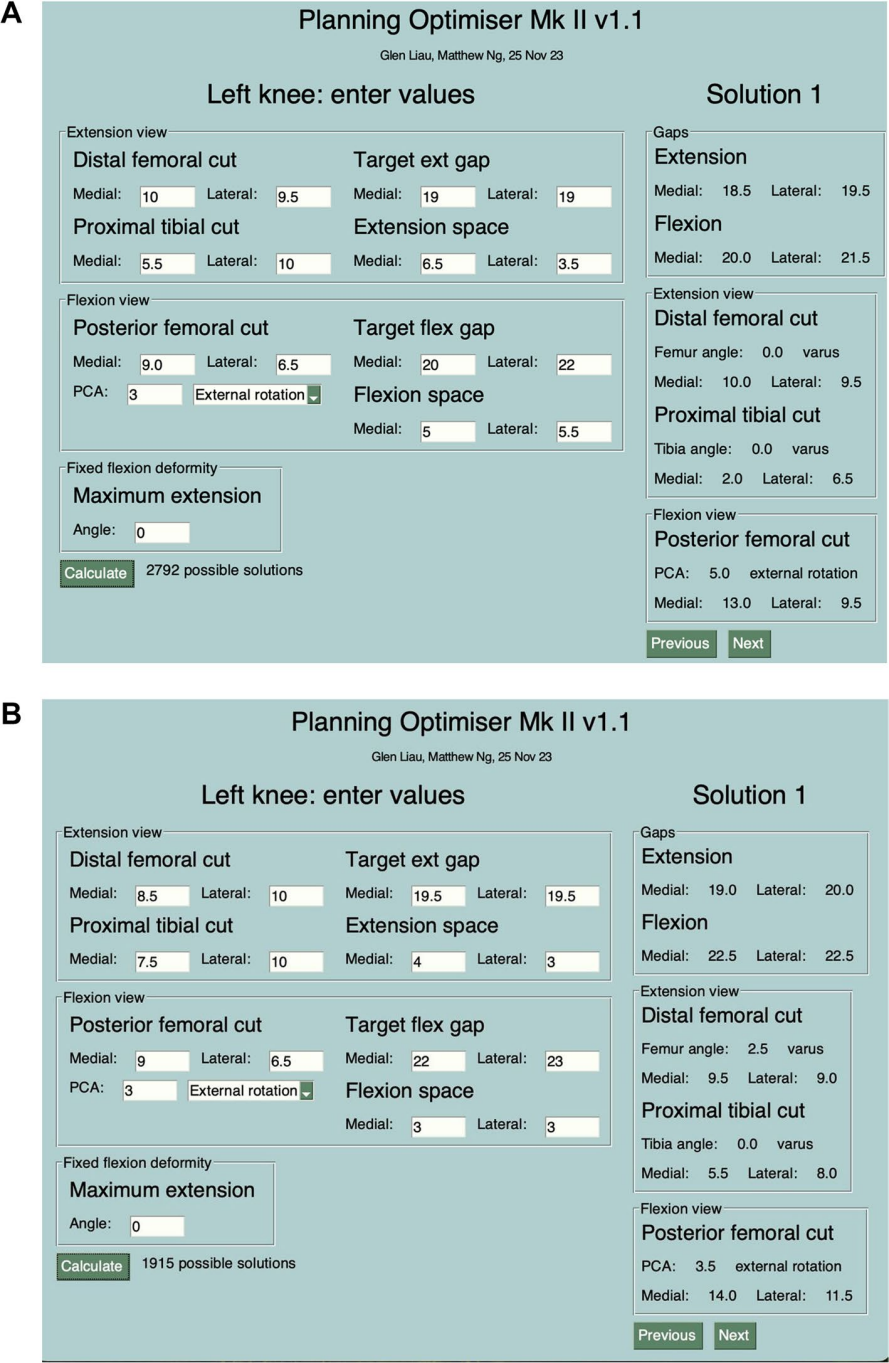
Screengrab of the Novel Algorithm for Balancing in rTKAs showing solutions generated: (**A**) Example A; (**B**) Example B

**Fig. 4:**
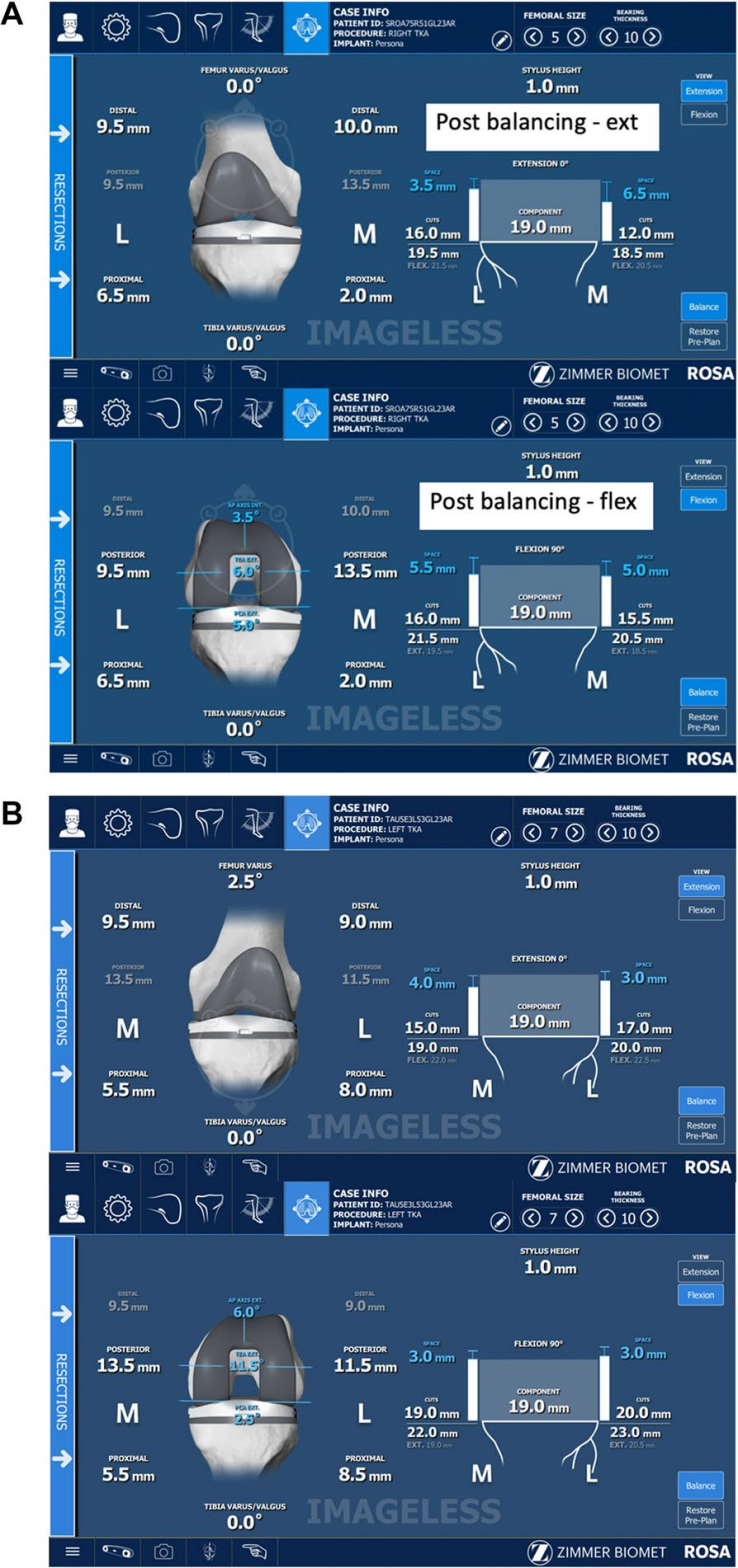
Post-Balanced rTKA using the Algorithm for (**A**) Example A (Top: Extension View; Bottom: Flexion View); (**B**) Example B. (Top: Extension View; Bottom: Flexion View)

Following the registration and calibration of the robot and determination of the knee position in 3D space, the surgery then enters the planning stage of the ROSA rTKA. In this phase, the ROSA display screen shows the initial state of the knee—particularly the initial a) medial & lateral distal femoral bone cuts (mm), b) medial & lateral posterior femoral bone cuts, c) medial & lateral proximal tibial bone cuts, d) initial posterior condylar axis state (degrees); and the corresponding initial e) medial & lateral flexion and extension spaces (mm), with consideration to the femoral component flexion/extension and tibial component slope degrees. These parameters are input into the algorithm, as well as the surgeon-defined target medial & lateral flexion and extension gaps (mm). This process is performed with the help of an assistant who is not scrubbed in (Fig. 3A and 3B). Tibial rotation data was not captured using the imageless ROSA system, and so tibial rotation was managed by all the surgeons intraoperatively using the Akagi line (Fig. 4).

The application, launched on an Apple MacBook, then runs the AI algorithm, which systematically translates and rotates the femoral and tibial implants in all possible planes of movement. It will collate the solutions that are within ± 0.5 mm of the surgeon-defined target gaps, and then rank all the solutions according to the surgeon’s preferred philosophy. “Solutions” refer to the set of specific rotations and translations of the femoral and tibial implants, and the distal femoral, posterior femoral, and proximal tibial bone cuts required to achieve the target surgeon plan. The application then displays the solutions in order of most to least preferred. All the above happens within less than 0.1 s of a button click. The initial parameters required for the algorithm to run are keyed into our application on the MacBook by an assistant who is not scrubbed in. Our application, which is independent of the ROSA console and not connected to it, displays the solutions and ranks them accordingly. The surgeon then selects their preferred solution. The chosen solution is then input into the ROSA console by the ROSA representative present in the operating theatre, as is standard practice in the institution, with or without the algorithm.

Instead of manually trialling and adjusting the femoral and tibial implants on the virtual display to achieve the surgeon’s preferred gap targets—based on individual tolerances and philosophies—our AI algorithm streamlines this process by providing the precise set of rotations and translations needed to achieve his/her desired flexion and extension gaps while accounting for the surgeon’s specific preferences and alignment principles. The solution can then be quickly reflected onto the virtual display and the plan confirmed.

The method by which the solutions are ranked can be easily customised via minor code adjustments to better suit the preferences and tolerances of each surgeon.

In the rare event where there is no possible solution, a pop-up will appear informing the surgeon that there are no solutions and to consider releases to balance the knee in accordance with their preferred alignment philosophy.

For this study, the algorithmized TKAs were performed by a single surgeon, using functional alignment philosophy principles. Non-algorithmized TKAs were performed by two separate surgeons, also according to functional alignment principles. To minimize reporting bias, surgeons in the non-algorithm group were blinded to the study's objectives and unaware that an algorithm was being used in other cases. To reduce selection bias, patients were selected as a consecutive series of primary knees with varus and valgus deformities based on their operation date. All three surgeons are colleagues of the same seniority working in the same tertiary referral institutions. All surgeons are board-certified arthroplasty attendings with 4 to 5 years of post-certification experience, including 3 to 4 years of experience utilizing the ROSA robotic system.

### Intraoperative process

All patients were operated on by surgeons trained in ROSA robot-assisted knee arthroplasty. The same surgical procedure was performed in all patients. After the tourniquet was applied, a medial parapatellar arthrotomy was performed with minimal soft tissue release during the initial exposure, mainly of the deep MCL in a varus knee. The femoral and tibial pins and trackers are then inserted, enabling the registration and determination of the 3D position of the knee. The algorithm was then utilised as described above, within the principles of functional alignment. The bone cuts and implants were then inserted accordingly. Femoral rotation in our study was determined using anatomical landmarks obtained through the robotic system’s (ROSA) registration process. Specifically, the posterior condylar axis, the transepicondylar axis, and the anteroposterior (Whiteside’s) line were registered intraoperatively and used to guide rotational alignment. No gap balancer tool was used. In the cases involved in the study, a CR liner was used as the first line of choice. If the PCL (Posterior Cruciate Ligament) was noticed to be functionally incompetent during the operation, an MC liner was utilised instead. No PS liners were used by the algorithm surgeon.

Minimal soft tissue release was performed initially during the exposure by all three surgeons, mainly of the deep MCL in a varus knee.

### Data extraction and outcomes

Our primary outcomes of interest were the proportion of rTKAs whose final gaps were within ± 1.5 mm of the surgeon-defined target gaps in the Algorithm Group compared to the Non-Algorithm Group (accounting for the systemic error of the ROSA Knee System) [18], and the average deviation (mm) from the surgeon-defined target gaps between the two groups, for each gap (Medial Extension, Lateral Extension, Medial Flexion, or Lateral Flexion Gaps).

As a secondary outcome, the average intraoperative planning duration of implant positioning and total surgical duration (minutes) were compared between the two groups.

An in-vivo study by Rossi and colleagues investigated the differences between the planned resections and final performed resections (performed resections were determined manually using callipers), to determine the accuracy of the bone cuts in this imageless robotic system to achieve planned resections [18]. They found that there existed a 1–2 mm error or discrepancy in the planned cut on the robot system, and the final performed cut, depending on the specific cut (distal, posterior femoral, or tibial cut) [18]. Therefore, a 1.5 mm deviation from the target was selected as the limit for an “accurate” soft tissue balancing to better account for the systemic errors regarding bone resection. Moreover, better clinical scores were achieved when the mediolateral gap difference was 2 mm or less [19, 20]. Thus, a deviation of more than 1.5 mm from surgeon-defined target gaps would likely result in a mediolateral gap difference of more than 2 mm.

To measure the difference in soft tissue balancing accuracy with our algorithm, before each case, the surgeon-defined target medial and lateral gaps were obtained from the surgeon himself. After each case, the final medial and lateral gaps were obtained directly from the ROSA Knee system records, which were evaluated after the final implants were implanted into the knee.

Our study evaluated both overall and individual soft tissue balancing accuracy. For overall soft tissue balancing accuracy, we first calculated the mean deviation of the final gaps for each case by summing the deviations of the medial extension, lateral extension, medial flexion, and lateral flexion gaps and dividing by four. A case was considered to have achieved accurate soft tissue balancing if this mean deviation was within ± 1.5 mm. We then determined the proportion of cases meeting this criterion in both the algorithm and non-algorithm groups.

Similarly, for individual soft tissue balancing accuracy, we assessed whether each gap (medial extension, lateral extension, medial flexion, or lateral flexion) met the surgeon-defined target. A gap was considered accurately balanced if the deviation from the target was within ± 1.5 mm. We then calculated the proportion of cases that met this threshold for each gap. Additionally, for each of the gaps, we quantified the average deviation from the target gap by summing the absolute deviations across cases and dividing by the total number of cases.

To measure the difference in surgical duration with our algorithm, the time taken for each case was obtained using the in-built electronic timer in the ROSA Knee system. We defined planning duration as the time from when the ROSA planning screen appears—following the assessment of initial knee gaps and laxities through knee ranging and varus-valgus stress testing—until the surgeon finalizes the implant position and proceeds with the first bone cut. The total surgical duration was defined as from the beginning of registration till once the final bone cut was made. This would include time needed for the occasional need for repeated additional releases, re-cuts, and re-balancing after initial planning. In doing so, we eliminated other factors as much as possible that might differ from patient to patient, or surgeon to surgeon, such as time for anesthesia, time taken for skin and soft tissue incisions, exposures, cementing time, washout time, and time taken for closure.

### Data analysis

All statistical analyses were conducted using RStudio (Version 2022.12.0 + 353). The means and standard deviations were computed for all continuous variables. The Shapiro-Wilks Test was used to determine normality of the various demographic and outcome data. Chi-square tests were used for discontinuous variables, while T-tests were used for continuous variables. Statistical significance was defined as *P* < 0.05. No missing data were encountered throughout the study. To determine the required sample size for our study, a power analysis was conducted. The analysis indicated that 44 patients are required to achieve a significance level of 0.05 with a power of 0.80.

Ethics approval was obtained from the local National Healthcare Group (NHG) Domain-Specific Review Board (2023/00955).

### Demographics

A total of 67 patients had undergone rTKA by three fully-qualified arthroplasty surgeons at our institution between November 2021 and December 2023. Out of the 67 patients, 25 belonged to the algorithm group as they had our algorithm utilised intra-operatively, while 42 patients belonged to the non-algorithm group as the algorithm was not utilised for them. In the algorithm group, the mean age was 70.4 years ± 7.3, while in the non-algorithm group, the mean age was 70.5 years ± 6.9. 44% of patients in the algorithm group were men, and that was 42.9% in the non-algorithm group. Table 1 below summarises relevant demographics.

**Table 1:** Demographics. Statistical significance is defined as a *P*-value of < 0.05

	**Algorithm**	**Non-Algorithm**	***P*-Value**
Age (y)	70.4 years ± 7.3	70.5 years ± 6.9	0.6
Male (%)	44.0%	42.9%	0.8

## Results

### Overall soft tissue balancing accuracy

#### Proportion of cases achieving surgeon-defined targets

For the rTKAs in the algorithm group, 92% achieved the surgeon-defined target gaps ± 1.5 mm, significantly greater than the 52% of rTKAs in the non-algorithm group (*P* < 0.003) (Table 2).

**Table 2:** Overall Soft Tissue Balancing Accuracy

	**Algorithm Group**	**Non-Algorithm Group**	*P* value
% Achieving Target Gaps (± 1.5 mm)	92%	52%	*P* < 0.003
Average Difference from Target Gaps to Final Gaps (mm)	1.1 ± 0.5	1.8 ± 1.0	*P* = 0.003

#### Average deviation of final gaps from target gaps

The average difference between surgeon-defined target gaps and final achieved gaps was 1.1 mm ± 0.5 in the algorithm group, significantly lower than that in the non-algorithm group, which was 1.8 mm ± 1.0 (*P* = 0.003).

### Individual soft tissue balancing accuracy

#### Proportion of cases achieving surgeon-defined targets

For the medial extension gap, 92% of cases using the algorithm achieved the surgeon-defined target gaps ± 1.5 mm, compared to 62% without the algorithm, with a significant *P*-value of 0.02. The lateral extension gap had 87% of cases within 1.5 mm using the algorithm and 43% without, with a significant *P*-value of < 0.001 (Table 3).

**Table 3:** Proportions of Final Gaps within 1.5 mm of Surgeon-Defined Target Gaps

Gaps	Medial Extension Gap		Lateral Extension Gap		Medial Flexion Gap		Lateral Flexion Gap	
	**Algorithm**	**Non-Algorithm**	**Algorithm**	**Non-Algorithm**	**Algorithm**	**Non-Algorithm**	**Algorithm**	**Non-Algorithm**
Proportion (%) of rTKAs within 1.5 mm of Surgeon-Defined Target Gaps	92%	62%	87%	43%	95%	70%	95%	50%
*P*-Value	*P* = 0.02		*P* < 0.001		*P* = 0.04		*P* < 0.001	

For the medial flexion gap, 95% of cases using the algorithm were within 1.5 mm, compared to 70% without, with a significant *P*-value of 0.04. The lateral flexion gap showed 95% of cases within 1.5 mm using the algorithm and 50% without, with a significant *P*-value of less than 0.001.

#### Average deviation of final gaps from target gaps

For the medial extension gap, the average deviation was 1.0 ± 0.3 mm using the algorithm, compared to 2.3 ± 1.0 mm without the algorithm, with a statistically significant *P*-value of 0.002. The lateral extension gap showed an average deviation of 1.3 ± 1.1 mm with the algorithm and 3.44 ± 2.1 mm without, with a significant *P*-value of 0.01 (Table 4).

**Table 4:** Mean deviation of final gaps from Surgeon-Defined Target Gaps (mm)

Gaps	Medial Extension Gap		Lateral Extension Gap		Medial Flexion Gap		Lateral Flexion Gap	
	**Algorithm**	**Non-Algorithm**	**Algorithm**	**Non-Algorithm**	**Algorithm**	**Non-Algorithm**	**Algorithm**	**Non-Algorithm**
Average Deviation of Final Gaps from Target Gaps (mm)	1.0 ± 0.3	2.3 ± 1.0	1.3 ± 1.1	3.4 ± 2.1	1.1 ± 0.3	2.0 ± 0.5	1.2 ± 0.6	2.3 ± 1.0
*P*-Value	*P* = 0.002		*P* = 0.01		*P* = 0.04		*P* < 0.001	

For the medial flexion gap, the average deviation was 1.1 ± 0.3 mm with the algorithm and 2.0 ± 0.5 mm without, with a significant *P*-value of 0.04. The lateral flexion gap had an average deviation of 1.2 ± 0.6 mm with the algorithm and 2.3 ± 1.0 mm without, with a significant *P*-value of less than 0.001.

### Planning duration and surgical duration

The intraoperative planning duration for implant positioning was significantly shorter for the rTKAs in the algorithm group, with a mean duration of 1.16 min ± 0.11, compared to 14.5 min ± 8.3 in the non-algorithm group (*P* < 0.0001). Total surgical duration was also significantly lower for the rTKAs in the algorithm group, with a mean total surgical time of 38.4 min ± 14.9, compared to 73.7 min ± 19.6 in the non-algorithm group (*P* = 0.0002) (Table 5).

**Table 5:** Planning Duration and Total Surgical Duration

	**Algorithm Group**	**Non-Algorithm Group**	*P* value
Intraoperative Planning Duration (min)	1.16 ± 0.11	14.5 ± 8.31	*P* < 0.0001
Total Surgical Duration (min)	38.4 ± 14.9	73.7 ± 19.6	*P* = 0.0002

## Discussion

**Fig. 5:**
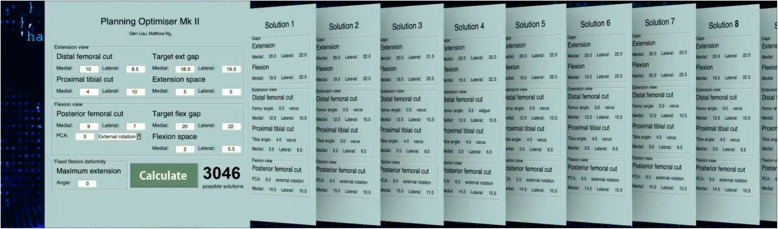
Graphical Depiction of the Multitude of Possible Solutions provided to the Surgeon. (Authors’ Own)

The principal finding of the study is that the in-house, novel algorithm developed improves soft tissue balancing accuracy and operative duration. This study presents a computerized algorithm utilized in the planning stage of ROSA rTKAs that automates the soft tissue balancing process. The algorithm requires input of the initial knee state parameters, such as the initial bone cuts, posterior condylar axis state, and extension spaces, followed by the surgeon’s target gaps. Running the algorithm then allows for almost the entire set of potentially thousands of solutions to achieve the surgeon’s targets from the initial state to be calculated in 0.1 s or less (Fig. 5).

Previously, the surgeon would manually rotate the virtual implants on the display screen and observe the changes to the hypothetical gaps until he or she was satisfied that the hypothesised gaps match his or her target extension and flexion gaps for the patient, before proceeding to perform the bone cuts.

In the algorithm group, the overall accuracy in terms of achieving the surgeon-defined target gaps was almost twice that without the algorithm. Moreover, both planning duration and overall surgical duration of the rTKA were significantly reduced.

An important consideration was the limit of the deviation from the target gaps by which a final gap was still considered “accurate”. There remains limited literature regarding how much discrepancy from the surgeon’s target extension and flexion gaps with respect to the final gaps can be accepted. This was chosen as 1.5 mm in the present study to reflect and account for the systemic error within the ROSA system regarding bone resections, as proven by Rossi et al. [18] Hypothesised reasons for the error include the thickness of the saw blade and the rough bone surface influencing the measure via the flat validation tools/callipers [18]. Nonetheless, work by Shalhoub and colleagues reflected similar sentiments in a separate imageless robotic total knee system, where they found the root mean square error between the predicted gaps by the system and measured final postoperative gaps was also approximately 1.5 mm in extension and flexion. [21]

A balanced knee is vital in preventing early postoperative instability, aseptic loosening, and early component wear [22]. Recently, it has been suggested that symmetrical flexion and extension gaps do not necessarily result in a balanced knee, and selected target gaps should be personalised to each patient’s anatomy and soft tissue tension [23, 24]. These nuances in achieving a balanced knee emphasise the importance of accurate balancing in accordance with surgeons’ targets.

However, with multiple degrees of freedom in terms of rotation of implants—specifically a) varus-valgus rotation of femoral component in extension, b) varus-valgus rotation of the tibial component in extension, c) proximalisation-distalisation of the femoral component in extension, d) proximalisation-distalisation of the tibial component in extension, e) external-internal rotation of the femoral component in flexion, f) anteriorisation-posteriorisation of the femoral component in flexion, g) flexion of the femoral component in sagittal view, h) extent of the posterior tibial slope in sagittal view—there are thousands of solutions possible for each case to achieve the surgeons’ targets. By manually rotating the components and observing the hypothetical gaps, it is difficult to achieve the surgeons’ targets in accordance with their philosophies and within their preferred boundaries in terms of component translation and/or rotation, which vary from surgeon to surgeon. This manual planning process is also time-consuming, which reduces the efficiency of the surgery.

The analysis of the accuracy in meeting each gap target reinforces the effectiveness of the AI algorithm. Across all four key gaps for balancing a knee in robotic TKA, the use of the AI algorithm resulted in significantly smaller deviation from surgeons’ targets compared to without the use of the algorithm, and increased soft tissue balancing accuracy for all gaps. For all the gaps, the proportions of accurate soft tissue balancing with our algorithm were steadily over 85%. Such results do suggest that the computerized algorithm developed is potentially a useful tool in enabling surgeons to achieve their preferred philosophy in ROSA rTKAs with increased efficiency and accuracy. In the soft tissue balancing process, the aforementioned challenge has been documented in literature, although no concrete solution has been developed presently [25]. We postulate that without the algorithm, surgeons may not always be able to manually adjust the components across all degrees of freedom to achieve their target gaps within the constraints of their alignment philosophy, especially given the time pressures of the operating theatre. As a result, they may settle for alternative final gaps that may not align optimally with their original surgical plan.

The algorithm surgeon’s practice is to plan for a slightly tighter gap in the compartment that has more osteophytes. For example, in varus knees, osteophytes predominantly form around the medial compartment, contributing to medial tightness. Once these osteophytes are resected during surgery, the medial soft tissues tend to stretch out more, leading to a medial gap that is slightly larger than initially planned [26, 27]. To account for this expected increase in the medial gap post-resection, it may be prudent to plan for a slightly tighter medial gap relative to the lateral gap during the initial planning phase. This strategy helps ensure more balanced flexion and extension gaps following bony and osteophyte resections. This is not a universally mandated standard but rather a surgical consideration based on the patient’s specific anatomy, intraoperative findings, and the surgeon’s preference. This is a demonstration of the uniqueness of this algorithm; it allows the surgeon to dial in his preferred targets and preferences on the fly, both pre-operatively and intra-operatively.

To our knowledge, only one other study by Young and colleagues presents the use of a computerised algorithm that can rapidly generate and ranking of thousands of possible solutions for positioning rTKA implants [25]. Young also recognised the pertinent issue that there can be thousands of possible solutions for a single rTKA case, depending on the surgeon’s preferred target flexion and extension gaps, the surgeon’s preferred alignment philosophy, and their preferred boundaries in terms of translation and/or rotation for each component [25]. Hence, they created an algorithm that was used on the MAKO Total Knee system with good effect, showing that while only 5% of all rTKAs were already balanced from the initial planning stage, 88% of their rTKAs could be balanced using the output from their algorithm [25].

However, their study has only been performed virtually and not clinically, based on values obtained from a primary TKA database. Their self-professed limitation relied on a mathematical gap calculation method when making adjustments, and they did not clinically confirm that each solution provided by the algorithm would provide “balance” after a TKA was implanted. Without this crucial clinical evaluation, other variables post saw cuts, such as changes to the soft tissue envelope, would impact the accuracy of their bony cuts. We have discovered these factors and considered them as we have refined our algorithm accordingly to achieve our results. We also noted that their algorithm could only be used on the MAKO system, and understandably, without evaluation of clinical application, the authors were not evaluating if there was an increase in accuracy or a reduction in surgical duration using their algorithm.

Our universal algorithm has been developed for use across a wide variety of industry-leading robotic knee systems, including, but not limited to, ROSA (Zimmer), MAKO (Stryker), CORI (Smith and Nephew) and VELYS (Johnson and Johnson) systems, accounting for nuances in the various robotic operating systems and their respective knee implants.

The algorithm surgeon’s practice is to plan for a slightly tighter gap in the compartment that has more osteophytes. For example, in varus knees, osteophytes predominantly form around the medial compartment, contributing to medial tightness. Once these osteophytes are resected during surgery, the medial soft tissues tend to stretch out more, leading to a medial gap that is slightly larger than initially planned. To account for this expected increase in the medial gap post-resection, it may be prudent to plan for a slightly tighter medial gap relative to the lateral gap during the initial planning phase. This strategy helps ensure more balanced flexion and extension gaps following bony and osteophyte resections. This is not a universally mandated standard but rather a surgical consideration based on the patient’s specific anatomy, intraoperative findings, and the surgeon’s preference.

### Impact

Our study has multiple strengths. First, it represents the first study that features a computerised algorithm for the ROSA Knee system, and the first clinical study that has evaluated the effect of the algorithm in knee balancing, after the final knee implants have been placed. Second, while there are different alignment philosophies for TKAs, there is still no consensus on which is the best philosophy. To add to the assortment of differing philosophies, there exists considerable intra-observer surgeon variability in employing the steps to achieve a range of targets that he has in mind. To properly compare the clinical outcomes of different philosophies, our novel solution can be a useful tool to algorithmically apply and evaluate alignment philosophies in a consistent and reproducible manner.

Our team plans to explore broader clinical adoption, both in Singapore and internationally, by working with suitable partners to bring our artificial intelligence algorithm to more surgeons and patients worldwide.

### Limitations

One limitation is that this study is not a randomised controlled trial. However, to reduce selection bias, patients were selected as a consecutive series of primary knees with varus and valgus deformities based on their operation date. All three surgeons were colleagues of the same seniority working in the same tertiary referral institutions. While the surgeons knew they were participating in our research study, they were purposefully blinded to the aims of the study, hence reducing the risk of selection bias that could occur (for instance, choosing to use our algorithm on less complex rTKAs so that the surgical duration would be falsely lowered).

Secondly, tibial axial rotational and positioning data were not captured or accounted for using our AI algorithm for this imageless ROSA Knee System—this represents another degree of freedom for implant positioning. Nonetheless, all surgeons in the study manage the axial position of the tibial implants using the Akagi line, minimizing heterogeneity in this regard.

Another limitation is that the rTKAs in this study were not performed by a single surgeon, but by 3 surgeons. However, it is worth noting that all surgeons are board-certified arthroplasty surgeons who are well-versed in the ROSA Knee system.

Finally, post-resection gap measurements were not collected. The goal of our algorithm is to optimize the pre-resection planning phase of the operation. Post-resection gap values, either with trial implants or actual implants, may be confounded by multiple variables, including but not limited to, the technique of sawing, further soft tissue releases or osteophyte removal performed after sawing, use of cemented or cementless implants, and how the implants were fitted into the joint.

Further studies may be considered to assess the relationship between planned and achieved balance, accounting for these variables.

## Conclusion

In conclusion, our novel computerised algorithm for soft tissue balancing in rTKAs represents the first clinically applied algorithmic way of improving the accuracy of achieving the surgeon’s target extension and flexion gaps significantly. It also reduces the duration needed for intraoperative planning and reduces the overall surgical duration. This is the first study that demonstrates the usefulness of such an algorithm in terms of achieving both reproducibility and efficiency in rTKAs.

## Data Availability

All available data and material will be made available upon reasonable request.

## Abbreviations

AI: Artificial Intelligence
GUI: Graphical User Interface
HKA: Hip-Knee Angle
NHG: National Healthcare Group
rTKA: Robotic Total Knee Arthroplasty
TKA: Total Knee Arthroplasty
